# Wearable Sweat Biosensors for Inflammatory Biomarkers (CRP, IL-6 and TNF-α): A Systematic Review of Analytical and Clinical Evidence

**DOI:** 10.3390/bios16090521

**Published:** 2026-09-18

**Authors:** Raghad S. Alsharidah, Razique Anwer, Maysoon Anwar Abdelhamid, Eman Amer Alanazi, Farah Mfwadh Alanazi, Haneen Fahaid Alanazi, Jumanah Naif Alotaibi, Lama Abdulaziz Alshathri, Renad Ali Alshehri, Rania Alsuhibani, Raghad H. Majrashi

**Affiliations:** 1 College of Medicine, Imam Mohammad Ibn Saud Islamic University (IMSIU), Riyadh 13317-4233, Saudi Arabia; 445003088@sm.imamu.edu.sa (R.S.A.); 443012915@sm.imamu.edu.sa (M.A.A.); 445005018@sm.imamu.edu.sa (E.A.A.); 444006175@sm.imamu.edu.sa (F.M.A.); 445000838@sm.imamu.edu.sa (H.F.A.); 445000952@sm.imamu.edu.sa (J.N.A.); 443021381@sm.imamu.edu.sa (L.A.A.); 443013211@sm.imamu.edu.sa (R.A.A.); 445001922@sm.imamu.edu.sa (R.A.); raghad.h.majrashi@gmail.com (R.H.M.); 2 Department of Pathology, College of Medicine, Imam Mohammad Ibn Saud Islamic University (IMSIU), Riyadh 13317-4233, Saudi Arabia

**Keywords:** sweat biosensors, C-reactive protein, interleukin-6, tumor necrosis factor-α, wearable devices, infection detection

## Abstract

*Background*: Wearable sweat biosensors have emerged as a promising non-invasive approach for monitoring inflammatory biomarkers, offering an alternative to repeated blood sampling. This systematic review evaluated the current evidence on the detection of C-reactive protein (CRP), interleukin-6 (IL-6), and tumor necrosis factor-α (TNF-α) in sweat for infection and inflammatory disease monitoring. *Methods*: Following PRISMA 2020 guidelines (PROSPERO: CRD420261371221), PubMed/MEDLINE, Scopus, Web of Science, IEEE Xplore, and Google Scholar were searched through April 2026. Human studies measuring CRP, IL-6, or TNF-α in sweat using wearable or biosensor-based platforms were included. Risk of bias and certainty of evidence were assessed using a modified Newcastle–Ottawa Scale/JBI checklist and GRADE. *Results*: Thirteen studies (2020–2025, 5–80 participants) were included. CRP showed the most consistent sweat-serum agreement (r = 0.844) and good diagnostic performance in one inflammatory bowel disease cohort (AUC = 0.845). TNF-α demonstrated the highest diagnostic accuracy in a single longitudinal study (AUC = 0.962), whereas IL-6 showed moderate sweat-serum correlations (R^2^ = 0.60–0.72) but limited diagnostic discrimination. IL-6 and TNF-α were more consistently detected in passively collected eccrine sweat, although IL-6 was also successfully measured following pilocarpine iontophoresis in one study. Most studies had moderate methodological quality. *Conclusions*: Sweat-based measurement of CRP, IL-6, and TNF-α is analytically feasible for non-invasive inflammatory biomarker monitoring. However, current evidence remains limited by small, heterogeneous studies, underscoring the need for standardized protocols and larger prospective clinical validation.

## 1. Introduction

Early recognition of infection and systemic inflammation remains an important challenge in clinical practice. Delayed identification of inflammatory responses may result in disease progression, delayed treatment, prolonged hospitalization, and poorer clinical outcomes. In addition to acute infections, many chronic inflammatory disorders, including inflammatory bowel disease (IBD), liver cirrhosis, chronic obstructive pulmonary disease (COPD), and cardiovascular diseases require repeated monitoring of inflammatory activity to guide treatment decisions. Consequently, reliable biomarkers that can be measured repeatedly and non-invasively have become an area of increasing research interest.

Among the available inflammatory biomarkers, C-reactive protein (CRP), interleukin-6 (IL-6), and tumor necrosis factor-α (TNF-α) are widely used to assess inflammatory activity. CRP is routinely measured as an acute-phase protein, whereas IL-6 and TNF-α play central roles in regulating the inflammatory response and are frequently elevated during infection and immune-mediated diseases [1]. Although these biomarkers are well established in clinical practice, their measurement still depends primarily on blood sampling. Repeated venipuncture may be inconvenient for patients requiring frequent monitoring and is less suitable for continuous assessment in outpatient settings, critically ill patients, or individuals requiring long-term follow-up.

Recent advances in wearable biosensor technology have generated considerable interest in sweat as an alternative biological fluid for biomarker detection. Sweat can be collected non-invasively and continuously, making it an attractive medium for wearable sensing devices [2,3]. Unlike blood sampling, sweat collection causes minimal discomfort and may allow repeated or even real-time monitoring without interrupting routine patient activities. These advantages have encouraged the development of electrochemical, microfluidic, and field-effect transistor-based biosensors capable of directly detecting inflammatory proteins in sweat.

Several studies have demonstrated that CRP, IL-6, and TNF-α can be detected in human sweat using wearable platforms [2,3,4,5,6,7]. Some investigations have reported encouraging correlations between sweat and serum concentrations, while others have explored their potential applications in patients with IBD, liver disease, cardiovascular disorders, and acute infections [5,6,7,8]. However, the available evidence remains fragmented. Most published studies involve relatively small cohorts, different sweat collection methods, and various sensing platforms, making direct comparison difficult. In addition, many studies primarily evaluate analytical feasibility rather than clinical diagnostic performance.

An important methodological consideration is the method of sweat collection. Emerging evidence suggests that certain cytokines, particularly IL-6 and TNF-α, are more consistently detected in passively expressed eccrine sweat than in iontophoresis-induced sweat [4]. One exception was noted: Björkenheim et al. used pilocarpine iontophoresis with Macroduct collection and successfully measured sweat IL-6. However, no significant group difference was observed [8], suggesting the collection method may affect detectability more than absolute measurability. If confirmed in larger studies, this observation could have important implications for the future design and clinical implementation of wearable sweat biosensors. Likewise, differences in sensor architecture, analytical sensitivity, and study populations continue to contribute to substantial heterogeneity across the current literature.

To date, no systematic review has critically examined the available evidence by integrating the analytical performance of sweat-based biosensors with their clinical applicability, methodological quality, and certainty of evidence. Addressing these aspects is important for understanding the current state of the field and identifying the challenges that must be overcome before sweat-based inflammatory biomarkers can be incorporated into routine clinical practice.

Although infection monitoring provided an important motivation for this review, the available evidence also includes chronic inflammatory conditions and analytical validation studies in healthy participants, which were included to characterize biomarker detectability and platform performance.

In the context of this review, sweat functions as the biological sample matrix that carries endogenous inflammatory biomarkers to the sensing interface. The designation “biosensor” refers not simply to measurement in a biological fluid, but to the coupling of a selective biological recognition element, such as an antibody, aptamer, or other affinity receptor, with a physicochemical transducer that converts target-receptor interaction into a measurable signal. Accordingly, wearable sweat biosensing involves three interconnected components: the sweat biofluid and its pre-analytical characteristics, selective molecular recognition of the target biomarker, and signal transduction. These components collectively determine analytical performance and ultimately influence clinical interpretability.

The novelty of this systematic review lies not in proposing a new sensing platform, but in critically integrating three elements that are often considered separately: sweat as a biological diagnostic matrix, biosensor architecture and analytical performance, and the strength of clinical validation for CRP, IL-6, and TNF-α.

Therefore, this systematic review aimed to evaluate the current evidence on sweat-based detection of CRP, IL-6, and TNF-α using wearable and biosensor-based technologies. We summarize the available evidence on biosensor architecture and biorecognition, analytical performance, sweat-to-blood relationships, clinical applications, methodological quality, and current research gaps, with particular attention to their potential role in infection and inflammatory disease monitoring.

## 2. Materials and Methods

### 2.1. Protocol and Registration

This systematic review was designed and conducted in accordance with the PRISMA 2020 statement [9]. To enhance methodological transparency and minimize the risk of selective reporting, the review protocol was prospectively registered with PROSPERO (Registration No. CRD420261371221) before the commencement of the review.

### 2.2. Eligibility Criteria

Studies were selected according to predefined eligibility criteria based on the PICO framework. The study population included participants of any age with suspected or confirmed infection or systemic inflammatory conditions. Eligible studies investigated the measurement of CRP, IL-6, or TNF-α in sweat using wearable, biosensor-based, microfluidic, or electrochemical detection platforms and compared these measurements with conventional blood-based biomarkers (serum or plasma CRP, IL-6, and TNF-α) or established clinical diagnostic methods, where applicable. Studies enrolling healthy participants only were eligible when they provided analytical validation of a sweat biosensor for a target biomarker, as such data establish baseline detectability and reference performance necessary to interpret findings in diseased cohorts. Outcomes of interest included diagnostic accuracy (e.g., sensitivity, specificity, and correlation with blood biomarkers), analytical performance, and the potential clinical applicability of sweat biomarkers for early infection detection or inflammatory disease monitoring. Eligible study designs included randomized controlled trials, phase II and III clinical trials, prospective and retrospective observational studies, and clinical validation studies involving human participants. In this review, no randomized controlled trials or phase II and III clinical trials were identified that fulfilled the eligibility criteria.

Studies were included if they (i) measured CRP, IL-6, or TNF-α directly in human sweat rather than exclusively in blood or serum; (ii) involved human participants in clinical, observational, or in vivo study designs; (iii) were published as full-text primary research articles in English; (iv) investigated infection, inflammation, or immune-mediated inflammatory conditions; and (v) evaluated wearable, biosensor-based, microfluidic, or electrochemical sweat detection platforms. Studies were excluded if they (i) evaluated only non-target biomarkers (e.g., glucose, lactate, alcohol, IFN-γ, or electrolytes); (ii) measured target biomarkers exclusively in blood or serum without sweat analysis; (iii) were in vitro, benchtop, or animal studies without human participants; (iv) assessed only physiological parameters such as heart rate or temperature without molecular sweat biomarkers; (v) lacked accessible full-text articles; or (vi) focused on behavioral, neurological, or ageing-related conditions without an infectious, inflammatory, or immune-mediated context.

### 2.3. Search Strategy

A comprehensive literature search was conducted in PubMed/MEDLINE, Scopus, Web of Science, IEEE Xplore, and Google Scholar from database inception to April 2026. The search strategy was developed in accordance with the review objectives and the PICO framework, using a combination of Medical Subject Headings (MeSH) and free-text keywords related to sweat, inflammatory biomarkers (CRP, IL-6, and TNF-α), infection, inflammation, wearable biosensors, and microfluidic sensing platforms. Boolean operators (“AND” and “OR”) were used to combine search terms and optimize the retrieval of relevant studies. The search strategy was adapted to the indexing system of each database, and the complete search strings are provided in Supplementary Table S1.

To ensure comprehensive study identification, the reference lists of all eligible articles and relevant review papers were manually screened for additional studies that were not identified during the electronic database search.

### 2.4. Study Selection and Data Extraction

**Figure 1:**
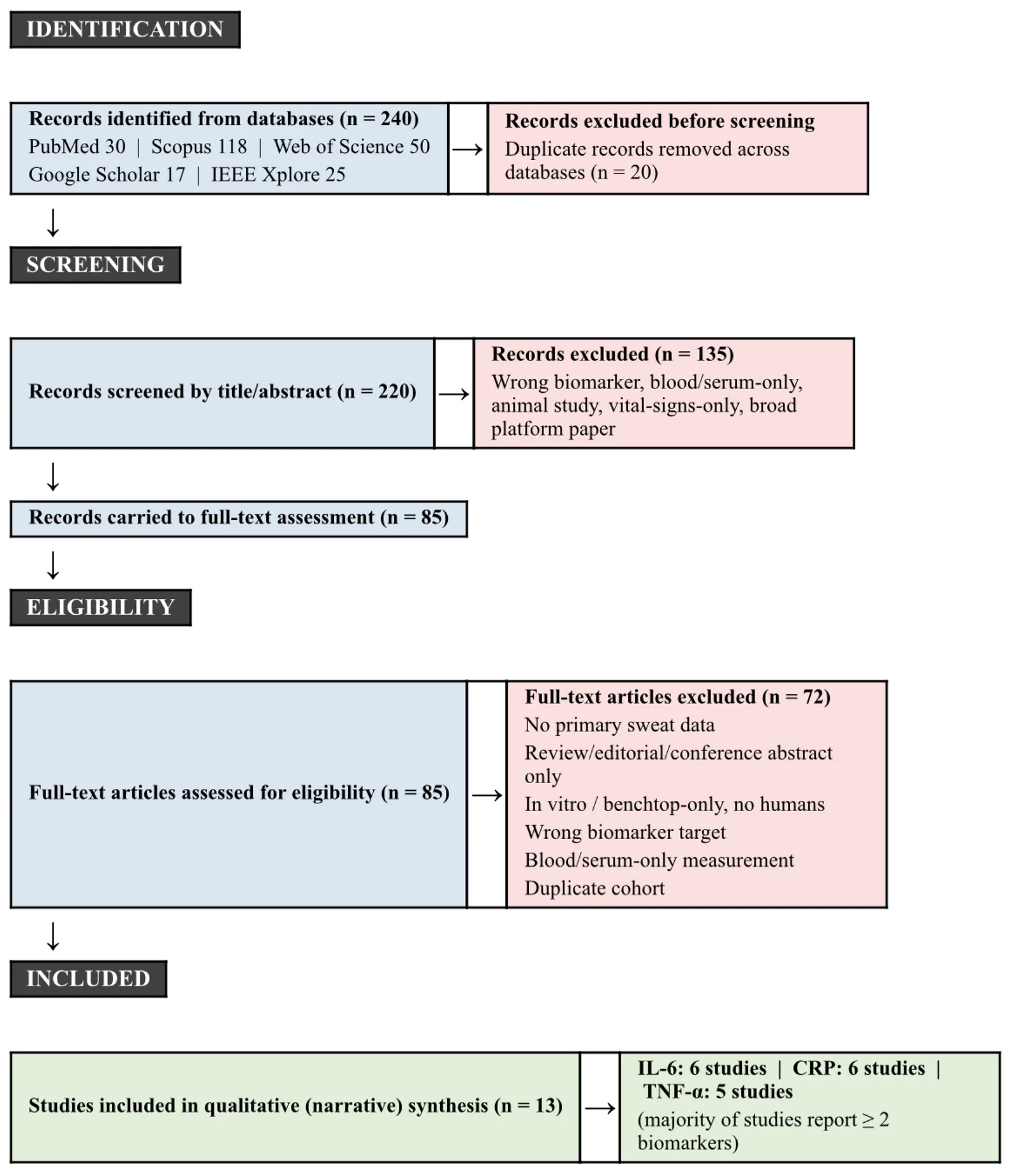
PRISMA 2020 flow diagram.

All retrieved records were imported into reference management software, and duplicate records were removed before screening. Two reviewers independently screened the titles and abstracts according to the predefined eligibility criteria. Full-text articles of potentially eligible studies were then retrieved and assessed independently for inclusion. Any disagreements were resolved through discussion, and where consensus could not be reached, a senior reviewer acted as the final arbiter. Reasons for exclusion at the full-text stage were documented and are summarized in the PRISMA 2020 flow diagram (Figure 1).

Data from the included studies were extracted independently by two reviewers using a standardized data extraction form developed a priori. The extracted information included study characteristics (title, first author, publication year, country, and study design), participant characteristics (population, sample size, age, sex, and health status), biomarker-related information (type of biomarker, biosensor platform, sweat collection method, and detection technique), comparator methods, and the principal study outcomes. All extracted data were cross-checked for accuracy, and any discrepancies were resolved through discussion among the reviewers.

### 2.5. Risk of Bias Assessment

The methodological quality of the included studies was independently assessed by two reviewers using a modified risk-of-bias assessment tool adapted from the Joanna Briggs Institute (JBI) Critical Appraisal Checklist [10] and the Newcastle-Ottawa Scale (NOS) for observational and clinical validation studies. The modified tool was selected to accommodate the methodological diversity of the included studies, particularly wearable biosensor validation studies that are not fully addressed by conventional appraisal instruments.

The assessment comprised 11 criteria across three methodological domains: Selection, Comparability, and Outcome/Exposure. Individual items were scored as 1 (criterion fulfilled) or 0 (criterion not fulfilled), yielding a maximum score of 11 points. Based on the total score, studies were classified as low risk of bias (≥8 points), moderate risk of bias (5–7 points), or high risk of bias (≤4 points).

Risk-of-bias assessment was performed independently by both reviewers, and any discrepancies were resolved through discussion until consensus was reached, with consultation from a senior reviewer when required.

A formal assessment of publication bias was not performed because no meta-analysis was undertaken, and the number of eligible studies was insufficient for reliable statistical evaluation.

### 2.6. Data Synthesis

A quantitative meta-analysis was not performed because the included studies differed substantially in study populations, biosensor platforms, sweat collection methods, target biomarkers, and outcome measures. Therefore, the findings were synthesized narratively by grouping studies according to the target biomarker (CRP, IL-6, and TNF-α). The evidence was critically compared with respect to analytical performance, sweat-blood correlation, diagnostic accuracy, clinical applications, and methodological quality.

### 2.7. Certainty of Evidence

The certainty of evidence was evaluated using the Grading of Recommendations Assessment, Development and Evaluation (GRADE) framework. Each outcome was assessed across the domains of risk of bias, inconsistency, indirectness, imprecision, and publication bias, and the overall certainty of evidence was rated as high, moderate, low, or very low. The assessment was performed independently by two reviewers, with disagreements resolved through discussion and consensus.

Deviations from the registered protocol: Minor deviations from the registered PROSPERO protocol (CRD420261371221) occurred during the conduct of this review. First, the risk-of-bias tool was changed from Newcastle-Ottawa/QUADAS-2 to a modified Newcastle-Ottawa/JBI checklist, as QUADAS-2 is designed for conventional diagnostic test accuracy studies and was found unsuitable for assessing the wearable biosensor validation studies included in this review. Second, Cochrane CENTRAL and the Cochrane Library were not searched, as preliminary scoping indicated these databases were unlikely to yield relevant records for this technical, device-focused literature, and the five databases searched (PubMed/MEDLINE, Scopus, Web of Science, IEEE Xplore, and Google Scholar) were judged sufficient to comprehensively capture the field. Third, quantitative meta-analysis was not performed, as originally planned, because the 13 included studies differed substantially in populations, biosensor platforms, sweat collection methods, and outcome measures, making statistical pooling inappropriate; findings were therefore synthesized narratively instead.

## 3. Results

### 3.1. Study Selection

A total of 240 records were identified through systematic searches of PubMed/MEDLINE (n = 30), Scopus (n = 118), Web of Science (n = 50), IEEE Xplore (n = 25), and Google Scholar (n = 17). Following removal of 20 duplicate records identified across databases, 220 unique records were screened by title and abstract, of which 135 were excluded for reasons including incorrect biomarker target, blood/serum-only measurement, animal studies, vital-signs-only focus, or being broad platform papers without primary biomarker data. The remaining 85 articles underwent full-text assessment according to the predefined eligibility criteria. Following full-text assessment, 72 reports were excluded according to the predefined eligibility criteria, and 13 studies were included in the qualitative synthesis. The study selection process is presented in the PRISMA 2020 flow diagram (Figure 1).

### 3.2. Characteristics of Included Studies

The 13 included studies, published between 2020 and 2025, reflect the growing body of research on wearable sweat biosensors for inflammatory biomarker detection, with the majority of studies (10/13) published between 2023 and 2025 [2,3,4,5,6,7,8,11,12,13,14,15,16]. Most studies were conducted in the United States (n = 8), followed by China (n = 4) and Sweden (n = 1) [2,3,4,5,6,7,8,11,12,13,14,15,16].

Sample sizes ranged from 5 to 80 participants [2,3,4,5,6,7,8,11,12,13,14,15,16], with most studies enrolling fewer than 30 participants, the largest cohort (n = 80) was reported by Tu et al. [6]. The included studies comprised prospective observational studies (n = 5), observational studies (n = 3), analytical or clinical validation studies (n = 3), and proof-of-concept studies (n = 2).

The study populations included both healthy volunteers (n = 6) and patients with IBD, liver cirrhosis, COPD, heart failure, ST-segment elevation myocardial infarction, active infection, post-COVID conditions, and atherosclerosis [2,3,4,5,6,7,8,11,12,13,14,15,16]. Sweat collection methods varied across studies, with passive eccrine sweat collection used most frequently (n = 6), followed by iontophoresis-induced sweat collection (n = 2), Macroduct-assisted collection (n = 2), exercise-induced sweating (n = 1), and automated sweat collection systems (n = 2) [2,3,4,5,6,7,8,11,12,13,14,15,16].

Among the evaluated biomarkers, CRP and IL-6 were each investigated in six studies, whereas TNF-α was evaluated in five studies. Several investigations simultaneously assessed two or more inflammatory biomarkers using multiplex wearable biosensor platforms. The detailed characteristics of the included studies are summarized in Table 1.

### 3.3. Sweat Biomarker Findings

#### 3.3.1. C-Reactive Protein (CRP)

CRP was evaluated in six of the 13 included studies, involving healthy participants as well as patients with IBD, liver cirrhosis, COPD, heart failure, active infection, and post-COVID conditions [2,3,5,6,7,13]. Reported sweat CRP concentrations ranged from 0.05–3 ng/mL in healthy individuals to 525–1175 pg/mL in patients with IBD, depending on the study population, sweat collection method, and analytical platform [3,5].

Three studies reported quantitative paired sweat-serum comparisons for CRP [5,6,7]. These were reported using statistics that are not directly equivalent and are therefore not pooled: Tu et al. reported a Pearson correlation of r = 0.844 [6], whereas Hirten et al. and Davis et al. reported coefficients of determination (R^2^ = 0.5278 overall, rising to R^2^ = 0.883 for serum CRP ≤ 10 µg/mL [5]; R^2^ = 0.392 across cirrhosis stages [7]). Jagannath et al. reported only a qualitative comparison, noting serum concentrations more than 100-fold higher than sweat, without a correlation coefficient [3]. Fu et al. reported the highest sensor-ELISA agreement (r = 0.961) using artificial CRP-spiked sweat samples, though without paired serum validation [13].

In the largest clinical study, Tu et al. evaluated 80 participants and reported a sweat-serum correlation of r = 0.844 using a wearable electrochemical patch with integrated pH and temperature compensation [6]. Hirten et al. reported a correlation of R^2^ = 0.5278, which increased to R^2^ = 0.883 among participants with serum CRP concentrations ≤ 10 µg/mL [5]. Jagannath et al. reported a correlation of r = 0.95 with a limit of detection of 1 pg/mL using the SWEATSENSER platform [2], while their subsequent study demonstrated a higher (less sensitive) detection limit of 0.2 ng/mL together with Bland-Altman agreement between sensor and reference-method CRP measurements in Macroduct-stimulated sweat [3]. Fu et al. reported excellent agreement between a wearable microfluidic ring sensor and ELISA (r = 0.961) with high analytical selectivity against other inflammatory cytokines [13].

Clinical studies also evaluated sweat CRP concentrations across different inflammatory conditions. Hirten et al. reported significantly higher sweat CRP concentrations in patients with inflammatory bowel disease than in healthy controls, with an AUC of 0.845, 82% sensitivity, and 70% specificity for disease discrimination [5]. Davis et al. observed increasing sweat CRP concentrations with worsening liver cirrhosis severity and reported moderate sweat-serum correlation across different disease stages [7]. Detailed CRP findings are summarized in Table 2.

#### 3.3.2. Interleukin-6 (IL-6)

IL-6 was evaluated in six of the 13 included studies, involving healthy participants as well as patients with IBD, liver cirrhosis, ST-segment elevation myocardial infarction (STEMI), and other inflammatory conditions [4,5,7,8,11,16]. Reported sweat IL-6 concentrations varied across study populations and analytical platforms, reflecting differences in biosensor technologies, sweat collection methods, and clinical characteristics.

Paired sweat–serum comparisons for IL-6 were reported in clinical studies. Hirten et al. reported an overall correlation of R^2^ = 0.60, which increased to R^2^ = 0.72 among participants with lower serum IL-6 concentrations [5]. 

Analytical validation studies demonstrated good agreement between wearable biosensors and conventional laboratory assays. Jagannath et al. reported excellent agreement between sweat IL-6 measurements and ELISA (r = 0.99), with analytical accuracy exceeding 90% and specificity greater than 95% [4]. Chu et al. developed a wearable fabric biosensor capable of detecting IL-6 with a reported limit of detection of 280 fg/mL across a broad analytical range [11]. Björkenheim et al. quantified IL-6 using the Olink^®^ Proximity Extension Assay and reported normalized protein expression (NPX) values rather than absolute sweat concentrations [8].

Clinical studies evaluated IL-6 across different inflammatory conditions. Hirten et al. reported an AUC of 0.62 for distinguishing patients with IBD from healthy controls using sweat IL-6 concentrations [5]. Davis et al. observed increasing sweat IL-6 concentrations with worsening liver cirrhosis severity [7], whereas Shahub et al. demonstrated continuous monitoring of IL-6 using a wearable device in which the removable sweat sensor connects to a wearable reader placed on the forearm during longitudinal assessment [16]. Jagannath et al. reported detectable IL-6 in passively collected eccrine sweat, whereas IL-6 was not readily expressed in iontophoresis-induced sweat [4]. In contrast, Björkenheim et al. successfully measured sweat IL-6 following pilocarpine iontophoresis with Macroduct collection, although no significant group difference was observed [8].

The principal findings for IL-6, including study populations, analytical methods, sweat concentrations, sweat-serum correlations, and diagnostic performance, are summarized in Table 3.

#### 3.3.3. Tumor Necrosis Factor-α (TNF-α)

TNF-α was evaluated in five of the 13 included studies, involving healthy participants as well as IBD, liver cirrhosis, and other inflammatory conditions [4,7,12,14,15]. Reported sweat TNF-α concentrations varied across study populations and biosensor platforms, reflecting differences in analytical methods and clinical settings.

Direct sweat-serum correlation data were reported in a limited number of clinical studies. Hirten et al. reported the highest sweat-serum correlation (R^2^ = 0.72) in patients with IBD [12], whereas the remaining studies primarily focused on analytical validation of wearable biosensors without paired serum comparisons [14,15]. 

Analytical validation studies demonstrated good agreement between wearable biosensors and conventional laboratory methods. Jagannath et al. reported analytical accuracy exceeding 90%, specificity greater than 95%, and excellent agreement with ELISA using passively collected sweat samples [4]. Huang et al. developed regenerative field-effect transistor (FET)-based wearable biosensors capable of continuous TNF-α monitoring, reporting detection limits as low as 10 fM together with repeated sensor regeneration and stable analytical performance [14,15].

Clinical investigations also evaluated TNF-α in inflammatory disease settings. Hirten et al. reported an AUC of 0.962, with 90.9% sensitivity and 91.7% specificity for distinguishing patients with IBD from healthy controls using sweat TNF-α concentrations [12]. Davis et al. observed increasing sweat TNF-α concentrations across different stages of liver cirrhosis. The principal findings for TNF-α are summarized in Table 4.

### 3.4. Sweat Biofluid, Biorecognition, and Biosensor Transduction

The 13 included studies employed a range of wearable and laboratory-assisted biosensor platforms for sweat biomarker detection, with electrochemical sensing representing the predominant analytical approach [2,3,4,5,6,7,8,11,12,13,14,15,16]. Non-faradaic electrochemical impedance spectroscopy (nf-EIS) was the most frequently used sensing technique (7/13 studies), followed by square wave voltammetry (SWV) (2/13), differential pulse voltammetry (DPV) (1/13), field-effect transistor (FET)-based sensing (2/13), and proximity extension assay (PEA) (1/13). Reported analytical performance included limits of detection ranging from 10 fM for TNF-α to 8 pM for CRP and 280 fg/mL for IL-6, depending on the sensing platform and biomarker. Detailed characteristics of the wearable biosensor platforms and their analytical performance are summarized in Table 5.

### 3.5. Risk of Bias and Methodological Quality Assessment

Methodological quality was assessed using the modified Newcastle-Ottawa Scale (NOS)/JBI checklist, comprising 11 criteria across the domains of Selection, Comparability, and Outcome/Exposure. Individual study scores ranged from 4 to 7 (Table 6).

Of the 13 included studies, 12 were classified as having a moderate risk of bias (scores 5–7/11), whereas one study (Hirten et al. [5], CRP/IL-6 study) was classified as high risk of bias (score 4/11). No study achieved a low risk of bias rating (≥8/11). The highest methodological scores (7/11) were obtained by Chu et al. [11] and Jagannath et al. [3].

The most frequently identified methodological limitations included small sample sizes (5–80 participants, with most studies enrolling fewer than 30 participants), absence of assessor blinding, limited representative sampling, lack of disease comparator groups, and incomplete reporting of analytical parameters, particularly the limits of detection (LOD) and quantification (LOQ). Representative sampling was reported in only one study, whereas all included studies employed appropriate statistical analyses and satisfied the exposure assessment criteria. Detailed quality assessment scores are presented in Table 6.

### 3.6. Certainty of Evidence (GRADE)

The certainty of evidence was assessed using the GRADE framework across six pre-specified outcome domains. As all included studies were observational, the initial certainty of evidence was rated as low before assessment of the predefined GRADE domains.

Evidence for CRP detection accuracy and sweat-serum correlation was rated as low certainty, representing the highest certainty level among the evaluated outcomes. The certainty of evidence for IL-6 detection accuracy, TNF-α detection accuracy, clinical infection detection, and sensor analytical performance was rated as very low.

Downgrading of evidence was primarily based on risk of bias, inconsistency, indirectness, and imprecision across the included studies. Additional downgrading for suspected publication bias was applied to sensor analytical performance. Given that certainty of evidence was low-to-very-low across all outcomes, these findings should be regarded as hypothesis-generating rather than confirmatory. The detailed GRADE evidence profiles are presented in Table 7.

## 4. Discussion

This systematic review synthesized evidence from 13 studies published between 2020 and 2025 evaluating the detection of CRP, IL-6, and TNF-α in human sweat using wearable biosensor technologies. Overall, the findings demonstrate that inflammatory biomarkers can be measured in sweat using a range of electrochemical sensing platforms, although the level of clinical evidence differs substantially among biomarkers. CRP was supported by the largest body of evidence, with the most consistent sweat-serum correlation and the broadest evaluation across inflammatory diseases, including IBD, liver cirrhosis, COPD, heart failure, active infection, and post-COVID conditions [5,6,7,13]. In contrast, IL-6 demonstrated moderate agreement with serum concentrations and variable diagnostic performance across studies [4,5,11], while TNF-α showed excellent diagnostic accuracy in a limited number of clinical investigations but remains supported by a comparatively smaller evidence base [12,14,15]. Although TNF-α showed the highest reported diagnostic accuracy (AUC = 0.962) in a single clinical investigation, CRP has been evaluated across a broader range of analytical and clinical settings. These findings represent different dimensions of the evidence and do not establish superiority of one biomarker over another. Given the limited sample sizes and low-to-very-low certainty of evidence, neither biomarker can currently be considered clinically validated.

Beyond biomarker performance, this review identified important methodological considerations that may influence analytical accuracy. Among these, sweat collection methodology emerged as a key factor, particularly for cytokine detection, as IL-6 and TNF-α were consistently detected in passively collected eccrine sweat, whereas findings following iontophoresis-induced sweat stimulation were more limited: Jagannath et al. [4] reported that both cytokines were not readily expressed in iontophoresis-induced sweat, while Björkenheim et al. [8] detected sweat IL-6 via pilocarpine iontophoresis but found no significant difference between STEMI patients and controls. Considerable variation was also observed in biosensor architecture, analytical sensitivity, study design, and participant populations, limiting direct comparison between studies. Although wearable electrochemical biosensors demonstrated encouraging analytical performance, the certainty of evidence remained low for CRP-related outcomes and very low for all remaining outcomes, reflecting methodological limitations, small sample sizes, and limited independent clinical validation.

Among the three inflammatory biomarkers evaluated, CRP emerged as the most extensively evaluated candidate to date for sweat-based monitoring, supported by the largest body of evidence and the broadest evaluation across diverse inflammatory conditions; however, given the overall low certainty of evidence, this finding should be regarded as hypothesis-generating rather than indicative of established clinical utility. This observation is supported by both analytical validation studies and clinical investigations involving IBD, liver cirrhosis, COPD, heart failure, and infection [5,6,7,13]. Compared with IL-6 and TNF-α, CRP demonstrated the most reproducible sweat-serum agreement across independent studies, suggesting that it currently provides the most reliable indicator of systemic inflammatory activity in sweat. Among the three biomarkers evaluated, CRP has the broadest evidence base and the most frequently reported within-study sweat-serum associations. However, the low certainty of evidence, substantial cross-platform variability in absolute concentrations, and absence of validated sweat reference ranges preclude conclusions regarding clinical superiority or diagnostic readiness.

An important caveat concerns the interpretation of absolute sweat CRP concentrations. Reported values in healthy participants span a wider range (0.05–3 ng/mL) [3] than those reported in patients with IBD (525–1175 pg/mL) [5], and mean concentrations in healthy cohorts vary by more than two orders of magnitude across studies (11.6 pg/mL [2] versus 1835 pg/mL [7]), despite both being obtained with passively collected sweat. These discrepancies may reflect differences in platform calibration, sweat collection, sampling conditions, participant characteristics, and the absence of a common reference standard; the available evidence does not allow methodological and biological sources of variability to be disentangled. Consequently, absolute sweat CRP concentrations are presently interpretable only within a given platform, which remains a fundamental barrier to establishing diagnostic thresholds and tempers any conclusion that CRP is currently the most clinically applicable of the three biomarkers.

The comparatively stronger performance of CRP is biologically plausible. As a stable acute-phase protein synthesized predominantly by hepatocytes in response to IL-6 stimulation, circulating CRP concentrations remain elevated for longer periods during systemic inflammation than rapidly fluctuating cytokines [17,18]. This greater biological stability may contribute to the stronger agreement observed between sweat and serum CRP measurements and may also reduce the influence of transient physiological variation associated with sweat production. In contrast, cytokines such as IL-6 and TNF-α exhibit rapid temporal fluctuations, shorter circulating half-lives, and lower physiological concentrations, all of which may increase analytical variability when measured in sweat.

IL-6 demonstrated moderate sweat-serum agreement across the available studies but showed less consistent diagnostic performance than CRP. These findings suggest that IL-6 may be more suitable for monitoring dynamic inflammatory responses than for use as an isolated diagnostic biomarker. Importantly, the observation that IL-6 detection was inconsistent following iontophoresis-induced sweat stimulation highlights the potential influence of sweat collection methodology on cytokine recovery and reinforces the need for standardized sampling protocols in future investigations.

Although TNF-α was evaluated in fewer studies, it demonstrated the highest reported diagnostic accuracy in one clinical investigation. While these findings are encouraging, they should be interpreted cautiously because the current evidence remains limited to a small number of validation studies with relatively modest sample sizes. Consequently, larger multicenter investigations are required before the diagnostic performance of TNF-α can be independently established; neither TNF-α nor CRP can currently be recommended for routine clinical application.

One of the principal findings of this review is the rapid evolution of wearable biosensor technologies for inflammatory biomarker detection. Although several sensing strategies were identified, electrochemical platforms clearly dominate current research owing to their high analytical sensitivity, low power requirements, relatively simple miniaturization, and compatibility with continuous wearable monitoring [2,3,4,5,6,7,11,12,13,14,15,16]. These characteristics have positioned electrochemical biosensors as the leading technology for non-invasive inflammatory monitoring.

Despite the impressive analytical performance reported by several platforms, considerable heterogeneity remains in biosensor design, sensing principles, calibration procedures, and analytical reporting. Such variability complicates direct comparison between studies and limits the establishment of universally applicable performance benchmarks. Standardization of analytical validation, including consistent reporting of limits of detection, analytical range, recovery, repeatability, and agreement with reference laboratory methods, will therefore be essential for meaningful comparison of future biosensor platforms.

These findings suggest that the principal bottleneck in sweat biosensing may be shifting from analytical sensitivity alone toward pre-analytical and biointerface-related challenges. Several included platforms achieved very low detection limits, extending into the femtomolar range, yet substantial uncertainty remains regarding biomarker recovery from sweat, stability of the biorecognition interface within a variable biological matrix, and standardization of sweat collection and processing. Only a limited number of platforms incorporated matrix-compensation strategies, automated filtration, or correction for physiological factors such as pH and temperature, while the relationship between sweat and circulating biomarker concentrations remained incompletely characterized. Consequently, further progress in this field will depend not only on improving sensor sensitivity, but also on establishing reproducible approaches to sweat sampling, biomarker recovery, matrix compensation, and biointerface stability that permit reliable comparison across platforms and clinically meaningful interpretation of sensor outputs.

An additional finding emerging from this review concerns the influence of sweat collection methodology. Passive eccrine sweat collection consistently enabled cytokine detection, whereas iontophoresis-induced sweat showed limited performance for IL-6 and TNF-α [4], the single exception was Björkenheim et al., who measured sweat IL-6 following pilocarpine iontophoresis with Macroduct collection [8]. Although the underlying biological mechanisms remain incompletely understood, this observation suggests that pre-analytical factors may substantially influence biomarker recovery and should receive greater attention during biosensor development and clinical validation.

Finally, while several next-generation technologies, including FET-based sensors and microfluidic wearable devices, demonstrated remarkable analytical sensitivity, most remain at an early stage of clinical validation [14,15]. Consequently, future studies should focus not only on improving analytical performance but also on demonstrating reproducibility across independent clinical populations.

The findings of this review indicate that wearable sweat biosensors have progressed beyond proof-of-concept devices and are increasingly being evaluated in clinically relevant populations. Nevertheless, several challenges continue to limit routine clinical implementation. Among these, biological variability in sweat composition represents one of the most significant obstacles. Sweat production is influenced by environmental temperature, physical activity, hydration status, anatomical collection site, and individual physiological differences, all of which may affect biomarker concentration independently of disease activity [19].

Another important challenge is the absence of standardized clinical reference ranges for inflammatory biomarkers in sweat. Unlike serum CRP or cytokines, where clinically validated diagnostic thresholds are well established, equivalent reference values for sweat remain unavailable. Consequently, interpretation of absolute sweat biomarker concentrations remains difficult, particularly across different biosensor platforms and patient populations.

Clinical translation will also require rigorous multicentre validation studies involving larger and more diverse populations. Most studies included in this review enrolled relatively small cohorts and evaluated analytical performance under controlled research conditions. Demonstrating reproducibility across independent institutions, different healthcare settings, and broader disease spectra will be essential before sweat-based inflammatory biomarkers can be incorporated into routine clinical practice.

The findings of this review are consistent with the broader literature describing the rapid development of wearable electrochemical biosensors for health monitoring. Previous reviews have highlighted the analytical advantages of electrochemical sensing platforms, including high sensitivity, rapid response times, and compatibility with flexible wearable devices [20,21,22]. Although several sensing platforms have demonstrated favorable analytical performance, further clinical validation is required before their incorporation into routine practice [23,24,25,26,27,28].

The present review extends these observations by specifically evaluating inflammatory biomarkers associated with systemic inflammation and infection, while critically examining their analytical validation, sweat-serum agreement, and clinical applicability. 

More recent biosensor studies published after completion of several included investigations continue to demonstrate substantial improvements in analytical sensitivity, multiplex detection, and wearable device integration. Nevertheless, many of these studies remain limited to analytical validation or proof-of-concept evaluation, with relatively few reporting independent clinical validation against established blood-based reference standards. Consequently, although biosensor technology continues to advance rapidly, clinical evidence has developed more slowly than analytical innovation.

Several of the findings in this review align with broader evidence from the wider biomarker and wearable-sensing literature. The comparatively broader performance of CRP may partly reflect its well-characterized biology as a relatively stable acute-phase protein, whereas cytokine concentrations such as IL-6 and TNF-α can vary more dynamically during inflammatory responses. [29,30,31]. This broader clinical relevance of inflammatory biomarkers extends beyond sweat-based sensing: point-of-care biomarker testing is increasingly recognized as a means of accelerating early infection detection [32], fecal calprotectin remains an established marker for monitoring IBD activity [33], systemic inflammatory markers carry prognostic value in cirrhosis [34], CRP predicts exacerbation risk in COPD [35], inflammation is an emerging therapeutic target in heart failure with preserved ejection fraction [36], and circulating IL-6 has been associated with infarct size and adverse outcomes after ST-elevation myocardial infarction [37]. Collectively, these parallels support the biological plausibility of the biomarker patterns observed across the included sweat biosensor studies. Looking forward, advances in machine learning may improve the accuracy and interpretability of wearable sensor data [38], while economic evaluations will be essential to determine the cost-effectiveness of these technologies before broader clinical adoption [39].

## 5. Strengths and Limitations

This review has several strengths. To our knowledge, this is the first systematic review to comprehensively evaluate CRP, IL-6, and TNF-α as sweat-based inflammatory biomarkers while integrating evidence on biosensor technology, analytical performance, sweat-serum relationships, methodological quality, and certainty of evidence. The review was conducted according to PRISMA 2020 guidelines, prospectively registered in PROSPERO, and incorporated structured risk-of-bias and GRADE assessments to enhance methodological transparency.

Several limitations should also be acknowledged. The available evidence remains limited by relatively small sample sizes (ranging from 5 to 80 participants, with 11 of the 13 included studies [84.6%] enrolling fewer than 30 participants), heterogeneous study designs, variable sweat collection methods, and inconsistent analytical reporting across studies. Diagnostic accuracy estimates derived from such small, single-study cohorts should therefore be interpreted with caution and require validation in larger, adequately powered populations. Most biosensor platforms have undergone evaluation in single-center studies with limited independent replication, reducing confidence in their generalizability. Furthermore, substantial methodological heterogeneity precluded quantitative meta-analysis, and the review was restricted to English-language publications, introducing the possibility of language and publication bias.

## 6. Future Directions

Future research should prioritize large, multicenter prospective studies evaluating sweat biomarkers across diverse infectious and inflammatory diseases using standardized collection protocols and harmonized analytical methods. Establishing clinically validated reference ranges for sweat CRP, IL-6, and TNF-α should be considered a research priority, as these will be fundamental for translating biosensor measurements into clinically interpretable diagnostic information.

From a technological perspective, future wearable platforms should move beyond single-biomarker detection toward multiplex systems capable of simultaneously monitoring inflammatory biomarkers together with physiological parameters such as skin temperature, heart rate, and sweat rate. Integration of artificial intelligence and machine learning may further enhance the interpretation of continuous biomarker data, enabling personalized inflammatory monitoring and earlier recognition of disease progression.

Finally, future investigations should place greater emphasis on independent external validation, long-term device stability, regulatory requirements, cost-effectiveness, and real-world implementation. Addressing these challenges will be essential for translating wearable sweat biosensors from promising research tools into clinically adopted technologies for infection detection and inflammatory disease monitoring.

## 7. Conclusions

This systematic review demonstrates the analytical feasibility of measuring CRP, IL-6, and TNF-α in human sweat using wearable and biosensor-based platforms. However, current evidence is insufficient to establish clinical diagnostic utility because studies are small, heterogeneous, and supported by low or very low certainty of evidence. Future investigations should prioritize standardized sweat collection, paired sweat-blood validation, adequately powered prospective cohorts, and associations with clinically meaningful outcomes before routine clinical application can be considered.

## Figures and Tables

**Table 1:** Characteristics of Included Studies (n = 13).

#	Reference	Country	n	Population	Biomarkers	Study Design	Sweat Collection	Key Finding	Risk Level
1	[6]	USA	80	Healthy + COPD + HF + active infections + post-COVID	CRP	Observational + analytical validation	Iontophoresis-induced sweat (wearable patch)	Strong correlation between sweat and serum CRP (r = 0.844, n = 80). Elevated sweat CRP in COPD, HFpEF, active infections, and post-COVID subjects. LOD = 8 pM.	Moderate
2	[11]	China	5	Healthy subjects	IL-6	Observational clinical	Natural sweating during exercise (fabric-based wearable sensor)	Detection range of 1 pg/mL–100 ng/mL with an LOD of 280 fg/mL; high ELISA correlation	Moderate
3	[8]	Sweden	24	STEMI patients (n = 18) + controls (n = 6)	IL-6	Prospective single-center observational	pilocarpine iontophoresis with Macroduct sweat collector	Only sweat STAMBP was significantly elevated in STEMI patients vs. controls; sweat IL-6 showed no significant difference vs. controls and no significant temporal change (plasma IL-6 decreased significantly from acute phase to follow-up).	Moderate
4	[7]	USA	44	Healthy controls + cirrhosis (outpatients + inpatients)	CRP, IL-6 TNF-α	Prospective observational pilot cohort	Passive (arm-mounted wearable sensor, up to 3 days)	Sweat CRP, IL-6, and TNF-α correlated with serum levels. Patients with cirrhosis showed higher sweat biomarker levels than controls. Outpatients demonstrated evening/night peaks of inflammatory biomarkers. Elevated sweat IL-6 was associated with poorer transplant-free survival.	Moderate
5	[4]	USA	26	Healthy	IL-6, TNF-α	Pre-clinical validation + on-body testing (healthy subjects)	Passively expressed eccrine sweat (PharmChek FDA-approved patch on forearm)	Pearson’s r = 0.99 vs. ELISA; accuracy > 90%; specificity > 95%; sensor stability retained for 30 days post-lyophilization; mechanical stability demonstrated after 100 repeated motion cycles.	Moderate
6	[5]	USA	26	IBD (n = 16) + healthy (n = 10)	IL-6, CRP	Prospective observational pilot	Passive (antebrachial wearable device, up to 5 days	CRP AUC = 0.845, sensitivity 82%, specificity 70%; R^2^ = 0.88 at serum ≤ 10 µg/mL	High
7	[12]	USA	28	IBD (n = 16) + healthy (n = 12)	TNF-α	Prospective observational	Passive (wearable device, up to 5 days)	Sweat TNF-α levels can be measured longitudinally and correlate moderately with serum values (R^2^ = 0.72). Continuous mean sweat TNF-α levels were significantly higher in active IBD patients (2.11 pg/mL) than in healthy controls (0.19 pg/mL) (*p* < 0.0001). The device differentiated active IBD from healthy subjects with an AUC of 0.962	Moderate
8	[13]	China	10	Healthy subjects	CRP	Experimental wearable validation	Iontophoresis induced sweat (wearable ring sensor)	CRP r = 0.961 vs. ELISA; real-time non-invasive atherosclerosis monitoring	Moderate
9	[2]	USA	26	Healthy subjects	CRP	Preclinical validation study	Passive eccrine (PharmChek patch)	Mean sweat CRP 11.6 pg/mL; r = 0.95 vs. ELISA LOD =1 pg/mL	Moderate
10	[3]	USA	8	Healthy subjects	CRP	Preclinical observational	Iontophoresis (Macroduct)	LOD = 0.2 ng/mL, sweat CRP range 0.05–3 ng/mL, Bland–Altman bias = 1.34 ng/mL (n = 8/18)	Moderate
11	[14]	China	11	Healthy subjects	TNF-α	Proof-of-concept device validation	Automatic nanosphere array transport	The detection range for TNF-α protein in actual human sweat extends from 10 fM to 1 nM with a capability for at least 30 mechanical deformation cycles. TNF-α concentration in the blood of three volunteers is much higher than in their sweat under the same conditions, and one volunteer has nearly equal TNF-α concentrations in blood and sweat	Moderate
12	[15]	China	13	Healthy subjects	TNF-α	Proof-of-Concept device development	Self-driven sweat transport (wearable, volunteer site)	Key Finding: LFO-IFET enabled continuous real-time monitoring of TNF-α in untreated sweat, detection range 0.1 pM–1 nM. Device-ELISA agreement: r = 0.659 R^2^ = 0.95 Automatic sweat collection and filtration allowed direct sweat analysis without pretreatment.	Moderate
13	[16]	USA	12	IBD (n = 2) + healthy (n = 10)	IL-6	Prospective observational cohort	Passive (wearable sweat sensor with forearm reader)	Continuous sweat monitoring reveals diurnal expression of IL-6 and calprotectin, with calprotectin tracking elevated baseline inflammation in chronic disease and capturing real-time anti-inflammatory drug effects.	Moderate

Note: In Jagannath B et al. (2022), only 8 of the 18 enrolled participants were analyzed for CRP [3]. In Cong Huang et al. (2025), sample size varied by sub-experiment: n = 7 (PBS), n = 7 (artificial sweat), n = 13 (real sweat, dose-response), n = 12 (device–ELISA correlation, r = 0.659), and n = 1 (longitudinal ELISA validation) [15]. Hirten et al. reported R^2^ = 0.88 within the physiological range (serum CRP ≤ 10 µg/mL), whereas R^2^ = 0.5278 represents the overall unfiltered dataset [5].

**Table 2:** Sweat CRP Findings Across Studies.

Reference	n	Sweat CRP Concentration	Serum CRP	LOD	r/R^2^	Key Result
[6]	80	0–30 ng/mL	0–54 µg/mL	8 pM	r = 0.844	Elevated in COPD, HFpEF, and active infection; strong correlation with serum CRP
[5]	26	525–1175 pg/mL	>5 µg/mL (enrollment)	NR	R^2^ = 0.883	AUC = 0.845; sensitivity 82%; specificity 70%; IBD vs. healthy (*p* = 0.0062)
[7]	44	Control: 1835.50 pg/mL Inpatient: 2672.72 pg/mL (morning)	Inpatients: 2.60 mg/dL	NR	R^2^ = 0.392 (overall)	Higher sweat CRP in cirrhosis; significant serum correlation
[13]	10	0–100 ng/mL (Range)	NR	NR	r = 0.961	Accurate ring wearable sensor; strong ELISA agreement
[2]	26	11.6 pg/mL (mean) 9.33 pg/mL (median)	NR	1 pg/mL	r = 0.95	CRP detectable in healthy sweat and markedly lower than serum levels
[3]	8	0.05–3 ng/mL	>100× higher in serum than in sweat	0.2 ng/mL	NR	Non-invasive detection confirmed (Macroduct-stimulated sweat); Bland–Altman bias = 1.34 ng/mL

Note: LOD values are reported in the units used by the source publications (mass-based: pg/mL, ng/mL; or molar-based: pM) and are not directly interconvertible across studies without a defined molecular weight for the CRP species measured. Hirten et al. reported R^2^ = 0.88 within the physiological range (serum CRP ≤ 10 µg/mL), whereas R^2^ = 0.5278 represents the overall unfiltered dataset [5].

**Table 3:** Sweat IL-6 Findings Across Studies.

Reference	n	Sweat IL-6 Concentration	Serum IL-6	LOD	r/R^2^	Key Result
[11]	5	1 pg/mL–100 ng/mL (analytical range)	NR	280 fg/mL	NR	High ELISA correlation; healthy subjects only; fabric wearable platform
[4]	26	<12 pg/mL (healthy); 0.2–200 pg/mL (range)	NR	NR	r = 0.99 (vs. ELISA)	Accuracy > 90%; specificity > 95%; readily detected in passive sweat; limited expression following iontophoresis-induced sweat
[5]	26	0.45–3.14 pg/mL	NR	NR	R^2^ = 0.60 (all); R^2^ = 0.72 (≤10 pg/mL)	AUC = 0.62 (non-significant); serum-to-sweat ratio 36.27
[7]	44	Controls: 3.87 pg/mL Inpatients: 7.05 pg/mL	Inpatients: 24.5 pg/mL	NR	NR	Elevated in cirrhosis; associated with reduced transplant-free survival
[8]	24	NPX units only	Plasma IL-6 (NPX): “Elevated in STEMI”	NR	NR	sweat IL-6 showed no significant difference vs. control
[16]	12	Circadian pattern observed	NR	NR	NR	Disease-dependent diurnal patterns

**Table 4:** Sweat TNF-α Findings Across Studies.

Reference	n	Sweat TNF-α Concentration	Serum TNF-α	LOD	r/R^2^	Key Result
[4]	26	0.2–200 pg/mL Passively expressed sweat	NR	NR	r = 0.99	Accuracy > 90%; specificity > 95%; Bland-Altman bias +0.55 pg/mL; cytokine expression was limited following iontophoresis-induced sweat stimulation
[12]	28	IBD: 2.11 pg/mL; Healthy: 0.19 pg/mL	Daily serum values	NR	R^2^ = 0.72	AUC = 0.962; sensitivity 90.9%; specificity 91.7%; cutoff = 0.98 pg/mL; *p* < 0.0001
[7]	44	Controls: 4.92 pg/mL; Inpatients: 6.38 pg/mL; Outpatients: 6.01 ± 1.37 pg/mL	Inpatients: 11.02 ± 8.04 pg/mL	NR	NR	Higher in cirrhosis; correlated with serum levels
[14]	11	10 fM–1 nM	Higher in blood (3/4 volunteers)	10 fM	NR	Detection range for TNF-α protein in actual human sweat extends from 10 fM to 1 nM
[15]	13	19.07–22.88 pg/L	NR	NR	r = 0.659	LFO-IFET; real-time continuous monitoring; automatic sweat collection and filtration

Note: Reported as pg/L in the source publication [15]. The authors’ own molar conversion elsewhere in the same article (21.1 pg/L stated as equivalent to 1.2 pM) and the device’s reported detection range (0.1 pM–1 nM) both indicate that these values correspond to pg/mL (≈1.1–1.3 pM); the unit is reproduced here as published.

**Table 5:** Biosensor Architecture and Analytical Performance of Wearable Sweat Biosensing Platforms.

Reference	Target Biomarker(s)	Sweat Collection/Biofluid	Biorecognition Element	Transduction Mechanism	Platform	Analytical Performance
[6]	CRP	Iontophoresis-induced sweat; microfluidic routing	Anti-CRP capture antibodies conjugated to AuNPs on the graphene sensing electrode	Square-wave voltammetry/electrochemical immunosensing	Wireless InflaStat wearable patch	LOD 8 pM; sweat-serum r = 0.844; integrated pH/temperature calibration
[11]	IL-6	Natural/exercise-induced sweat	IL-6-specific aptamer functionalized on CNT/graphene fibers	Electrochemical aptamer sensing/SWV based on target-induced conformational change	Wearable electrochemical fabric	Range 1 pg/mL–100 ng/mL; LOD 280 fg/mL
[8]	IL-6	Pilocarpine-induced sweat collected using Macroduct	Paired oligonucleotide-labelled antibodies (Olink PEA)	Proximity extension assay with PCR-based readout	Olink Target 96 Inflammation (laboratory assay; non-wearable)	NPX units; no single quantitative sweat LOD reported
[7]	CRP, IL-6, TNF-α	Passive eccrine sweat; arm-mounted strip, up to 3 days	Target-specific monoclonal capture antibodies immobilized on the sensing interface	Non-faradaic electrochemical impedance spectroscopy (nf-EIS)	AWARE wearable	Significant sweat-serum associations; continuous monitoring of all three biomarkers
[4]	IL-6, TNF-α	Passive eccrine sweat; PharmChek-based sampling	Biomarker-specific monoclonal capture antibodies immobilized on the sensing electrodes	Non-faradaic electrochemical impedance spectroscopy (nf-EIS)	SWEATSENSER	r = 0.99 vs. ELISA; accuracy > 90%; specificity > 95%; stability up to 30 days
[5]	CRP, IL-6	Passive antebrachial sweat; up to 5 days	Monoclonal CRP and IL-6 capture antibodies immobilized on ZnO-based electrodes	Non-faradaic electrochemical impedance spectroscopy (nf-EIS)	IBD AWARE	CRP AUC = 0.845; CRP R^2^ = 0.883 within the specified serum range
[12]	TNF-α	Passive antebrachial sweat; up to 5 days	Monoclonal TNF-α capture antibody immobilized on the sensing interface	Non-faradaic electrochemical impedance spectroscopy (nf-EIS) at 180 Hz	IBD AWARE	R^2^ = 0.72; AUC = 0.962; sensitivity 90.9%; specificity 91.7%
[13]	CRP	Iontophoresis-induced sweat with microfluidic routing	CRP-specific aptamer anchored to the sensing interface; redox-reporter based	Electrochemical aptamer sensing/DPV	FredJewel wearable ring	CRP range 0–100 ng/mL; sensor-ELISA agreement r = 0.961
[2]	CRP	Passive eccrine sweat; PharmChek patch	Monoclonal CRP capture antibody immobilized on the affinity-functionalized electrode	Non-faradaic electrochemical impedance spectroscopy (nf-EIS)	SWEATSENSER	LOD 1 pg/mL; r = 0.95 vs. ELISA; approximately 3-log dynamic range
[3]	CRP	Pilocarpine-induced sweat; Macroduct for extracted CRP validation	CRP-specific monoclonal capture antibody on an affinity-functionalized sensing interface	Non-faradaic electrochemical impedance spectroscopy (nf-EIS)	SWEATSENSER	LOD 0.2 ng/mL; Bland-Altman bias 1.34 ng/mL
[14]	TNF-α	Automatically transported untreated sweat	TNF-α-specific aptamer on a functionalized MoS2 sensing surface	Field-effect transistor sensing	Nanosphere-MoS2 FET	Range 10 fM–1 nM; stable electrical performance after up to 30 extreme mechanical deformations
[15]	TNF-α	Self-driven collection and filtration of untreated sweat	TNF-α-specific nucleic-acid aptamer	Aptamer-modified ion-sensitive/FET electrical detection	LFO-IFET wearable	Range 0.1 pM–1 nM; r = 0.659 vs. ELISA; automated filtration
[16]	IL-6	Passive sweat; wearable forearm reader	Target-specific antibody-based affinity recognition interface	Non-faradaic electrochemical impedance spectroscopy (nf-EIS)	Two-plex SWEATSENSER	Continuous IL-6 monitoring; CV <= 11%; diurnal patterns observed

Note: Sweat serves as the biological sample matrix carrying endogenous target biomarkers to the sensing interface. In affinity-based biosensors, selective recognition is mediated by target-specific antibodies or aptamers, while electrochemical or field-effect transduction converts the molecular binding event into a measurable signal. Björkenheim et al. [8] employed a laboratory proximity extension assay rather than a wearable biosensor and is included because it provides relevant evidence on IL-6 measurement in sweat.

**Table 6:** Methodological Quality Assessment—Modified NOS/JBI (n = 13).

#	Reference	NOS/JBI Score	Risk Level & Key Limitations
1	[6]	6/11	Moderate—partial representative sampling; unclear outcome blinding; iontophoresis sweat (may underrepresent natural physiology)
2	[11]	7/11	Moderate—very small sample (n = 5); no paired blood sampling; healthy participants only; no follow-up
3	[8]	5/11	Moderate—small sample (n = 24); LOD calculated per-assay but not reported as a single numeric value
4	[7]	6/11	Moderate—pilot sample; predominantly male veterans; single-country; short monitoring (3 days); no formal sample size calculation
5	[4]	5/11	Moderate—partial sampling; no LOD stated for blood comparison; cross-sectional sick cohort; small sick group (n = 5)
6	[5]	4/11	High—partial sampling; hospitalized IBD only; no LOD/LOQ; single daily serum timepoint; controls recruited from different site
7	[12]	5/11	Moderate—partial sampling; no LOD/LOQ; no interference testing; controls from different site; 5-day monitoring period
8	[13]	6/11	Moderate—no representative sampling; no paired blood validation; healthy subjects only; single-country
9	[2]	6/11	Moderate—healthy cohort only; no paired blood sampling; no follow-up; conflict of interest noted
10	[3]	7/11	Moderate—healthy cohort only; small CRP sub-sample (n = 8); no blinding
11	[14]	5/11	Moderate—very small sample (n = 4–11); no statistical analysis; no correlation values; qualitative blood-sweat comparison only
12	[15]	5/11	Moderate—sample size varies by sub-experiment (n = 7–13); no LOD/LOQ; no paired blood validation (sweat-only comparison); limited demographic reporting
13	[16]	5/11	Moderate—very small disease cohort (n = 2 IBD); no paired blood validation; limited external clinical validation.

**Table 7:** GRADE Certainty of Evidence Profile Across Outcome Domains.

Outcome	Studies (n)	Risk of Bias	Inconsistency	Indirectness	Imprecision	Certainty Rating
IL-6 detection accuracy	6	Serious ↓	Serious ↓	Not serious	Serious ↓	VERY LOW
CRP detection accuracy	6	Serious ↓	Not serious	Not serious	Serious ↓	LOW
TNF-α detection accuracy	5	Serious ↓	Serious ↓	Not serious	Serious ↓	VERY LOW
Diagnostic accuracy for inflammation	3	Serious ↓	Serious ↓	Serious ↓	Very serious ↓↓	VERY LOW
Sweat vs. blood correlation	8	Serious ↓	Not serious	Not serious	Serious ↓	LOW
Sensor analytical performance	13	Serious ↓	Not serious	Serious ↓	Serious ↓	VERY LOW

## Data Availability

All data supporting the findings of this systematic review were derived from published studies. The extracted data and additional methodological information are provided in the Supplementary Materials.

## Supplementary Materials

The following supporting information can be downloaded at https://www.mdpi.com/article/10.3390/bios16090521/s1, Table S1: Full Search Strategies by Database; Table S2: Characteristics of Included Studies (n = 13); Table S3: Biomarker Quantification Details (Sweat vs. Serum); Table S4: Assessment—Modified Newcastle-Ottawa Scale/JBI Checklist; Table S5: List of Abbreviations; Figure S1: PRISMA 2020 Flow Diagram—Tabular Summary [40].

## Abbreviations

The following abbreviations are used in this manuscript:

**Abbreviation**	**Full Term**
CRP	C-Reactive Protein
IL-6	Interleukin-6
TNF-α	Tumor Necrosis Factor-alpha
LOD	Limit of Detection
LOQ	Limit of Quantification
IBD	Inflammatory Bowel Disease
ELISA	Enzyme-Linked Immunosorbent Assay
AUC	Area Under the Curve
EIS	Electrochemical Impedance Spectroscopy
nf-EIS	Non-faradaic Electrochemical Impedance Spectroscopy
COPD	Chronic Obstructive Pulmonary Disease
JBI	Joanna Briggs Institute
PRISMA	Preferred Reporting Items for Systematic Reviews and Meta-Analyses
GRADE	Grading of Recommendations Assessment, Development and Evaluation
FET	Field-Effect Transistor
SWV	Square Wave Voltammetry
STEMI	ST-Elevation Myocardial Infarction
IFN-γ	Interferon-gamma
TRL	Technology Readiness Level
CV	Coefficient of Variation
NOS	Newcastle-Ottawa Scale
IEEE	Institute of Electrical and Electronics Engineers
